# Topical Tacrolimus 0.1% in the Treatment of Thygeson’s Superficial Punctate Keratitis

**DOI:** 10.7759/cureus.101170

**Published:** 2026-01-09

**Authors:** Audrey-Anne Lapierre, Mélanie Hébert, René Dinh

**Affiliations:** 1 Department of Ophthalmology, Université Laval, Quebec, CAN; 2 Department of Ophthalmology, Hôpital du Saint-Sacrement, Quebec, CAN

**Keywords:** chronic keratitis, cornea pathology, immunomodulatory therapy, steroid-sparing treatment, thygeson’s superficial punctate keratitis, topical tacrolimus

## Abstract

Thygeson’s superficial punctate keratitis (TSPK) is a rare and chronic eye condition characterized by recurrent flare-ups of symptoms such as photophobia and tearing. Various treatments have been attempted over the years, but none have been entirely successful in managing the condition. This report presents the case of a 21-year-old woman with bilateral TSPK who exhibited photophobia, tearing, and burning sensations. The patient was treated with an off-label non-ophthalmic topical tacrolimus 0.1% ointment, applied nightly into the inferior fornix and titrated with artificial tears to improve tolerance. Complete resolution of corneal lesions and symptoms was observed after three weeks, so the treatment was stopped. Although lesions reappeared following cessation of treatment, symptomatic relief persisted. This case highlights the potential of non-ophthalmic tacrolimus 0.1% ointment, a higher-concentration formulation not previously described in the TSPK literature, as an effective therapeutic option that may contribute to prolonged symptom control while avoiding the intraocular pressure risks associated with steroid use, particularly in young patients. Tacrolimus may represent a promising alternative therapy for patients requiring chronic management of TSPK.

## Introduction

Thygeson’s superficial punctate keratitis (TSPK) is a chronic condition that mostly affects the superficial central cornea and is characterized by whitish-gray focal lesions within the corneal epithelium [1]. The distribution and quantity of lesions may vary over time. The diagnosis is clinical and confirmed based on patient history and the presence of superficial punctate corneal lesions, which stain positively with fluorescein. However, these can also stain negatively due to raised corneal lesions seen on slit-lamp examination [2]. Patients are typically young, otherwise-healthy individuals who present with symptoms of photophobia, tearing, burning sensations, pain, blurry vision, and eye redness during exacerbations. They typically experience minimal or no disturbance in VA [2].

The pathophysiology of this disease remains unclear, although a viral origin was initially suspected. A study demonstrated the absence of herpes simplex virus, varicella-zoster virus, and adenovirus in the corneal epithelium of patients with TSPK using polymerase chain reaction (PCR) [3]. Findings suggest that TSPK may instead be related to an immune-mediated mechanism, particularly due to its association with the human leukocyte antigen (HLA)-DR3 [4]. This genetic association may influence immune function and contribute to the recurrent exacerbations and remissions characteristic of the disease.

Various therapeutic approaches, including antiviral agents, topical corticosteroids, and cyclosporine, have been attempted, but none provide consistently effective long-term control, with some patients remaining refractory to these treatments [4]. Cyclosporine has been reported to reduce ocular inflammation; however, its effect may be limited by delayed onset of action, variable patient response, and the need for prolonged therapy [4]. Some patients require long-term topical corticosteroid use with recurrence upon tapering, but this brings side effects that may not be tolerated by some patients, including the risk of cataract development and steroid response-associated intraocular pressure (IOP) elevation [5], which may limit their use, particularly in younger patients. Lower-concentration tacrolimus formulations (0.02-0.03%) have also been reported to reduce ocular inflammation and improve symptoms, but their effect often requires extended treatment courses and has not been proven curative [6,7].

In this article, we present the use of topical non-ophthalmic tacrolimus 0.1% ointment for TSPK. Tacrolimus is a calcineurin inhibitor that may help reduce corneal inflammation by suppressing T-cell activity, thereby targeting the immune-mediated component of TSPK [4]. This higher-concentration formulation may provide a more potent and faster anti-inflammatory effect on both symptoms and lesions, offering a steroid-sparing alternative in TSPK management.

## Case presentation

**Figure 1:**
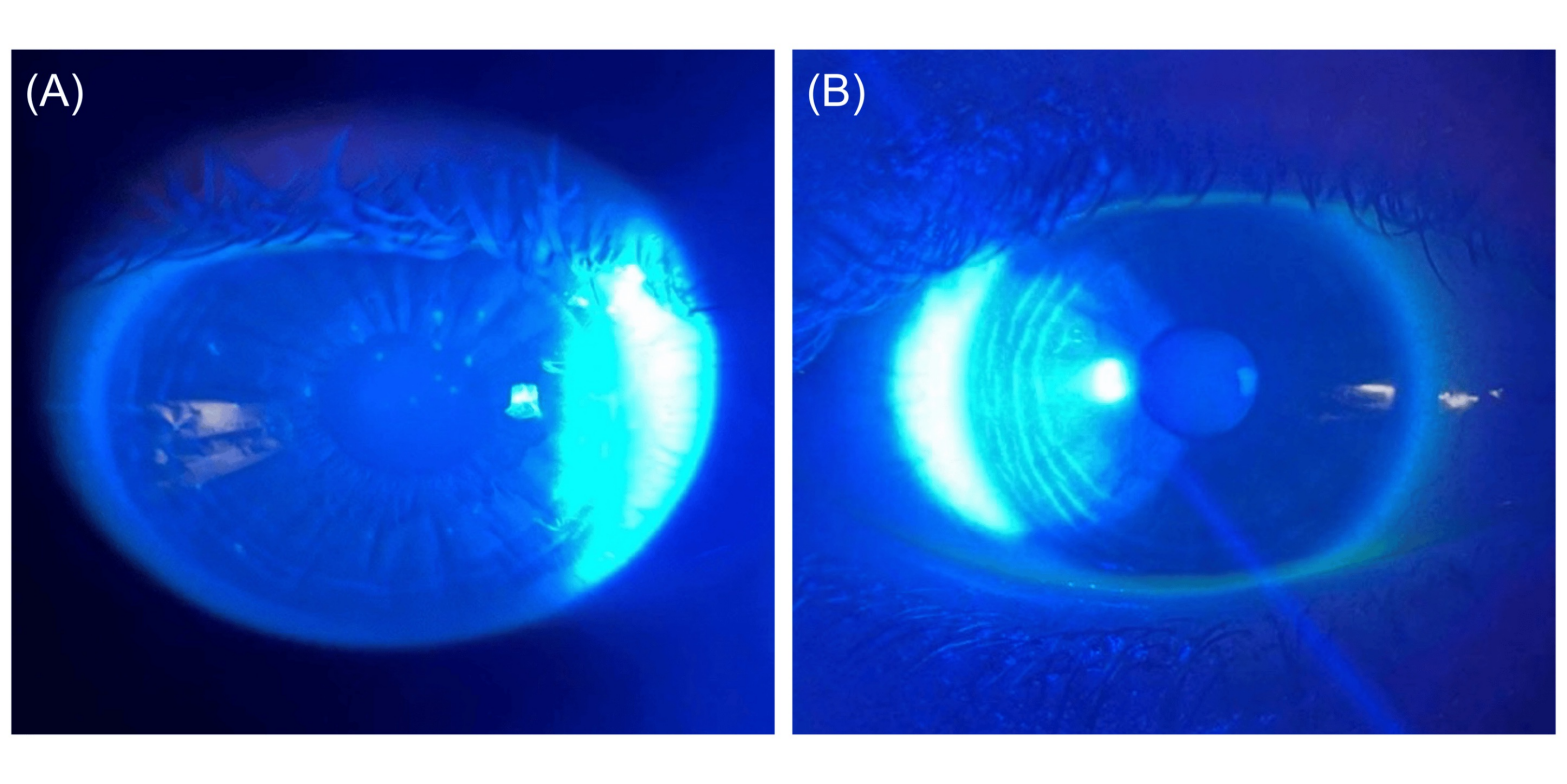
Slit-lamp photography of the cornea with fluorescein staining. (A) Several superficial elevated corneal lesions with positive fluorescein staining can be seen prior to the treatment of the right eye using tacrolimus. (B) No observable lesions were found after three weeks of treatment.

A 21-year-old woman with no other notable past medical history presented to the ophthalmology clinic with mild photophobia, tearing, and burning of the eyes, but no visual disturbances. Clinical examination revealed approximately 15-30 superficial, elevated, grayish-white corneal lesions in both eyes, consistent with classic bilateral TSPK (Figure 1A). The patient had only used artificial tears and did not receive first-line treatments such as topical corticosteroids, as she wished to avoid potential side effects and preferred an alternative treatment approach.

At presentation, she had a best-corrected Snellen visual acuity (VA) of 20/20 in both eyes (OU) and an IOP of 21 mmHg. The patient was prescribed topical non-ophthalmic tacrolimus 0.1% ointment, used off-label, to be applied twice daily into the inferior fornix. Artificial tears were continued as needed, but no other treatment was administered concurrently. The duration of treatment was three weeks. This decision was made in discussion with the patient, considering her preference to minimize prolonged topical therapy.

The patient reported a burning sensation with the first application of tacrolimus, along with temporary blurred vision due to the ointment’s consistency. As a result, the morning application was discontinued, and the ointment was applied only at bedtime. This bedtime-only regimen was followed consistently for three weeks and was well-tolerated.

At the three-week follow-up, the patient reported complete resolution of visual symptoms such as tearing, photophobia, and burning sensations. Best-corrected VA remained 20/20 OU, IOP was 17 mmHg OU, and the corneal lesions had completely resolved bilaterally (Figure 1B). After this period, the patient discontinued tacrolimus use entirely. At a two-month post-treatment follow-up, bilateral corneal lesions, which stained positively with fluorescein, had recurred; however, the patient remained asymptomatic, reporting no return of previous symptoms. As a result, it was decided not to resume tacrolimus treatment. At this visit, VA remained 20/20 OU, and IOP was 21 mmHg OU. At a follow-up visit 12 months later, the patient remained asymptomatic despite the return of stable corneal lesions, so the treatment was not restarted. She did not develop any complications, such as increased IOP or infectious keratitis. VA remained 20/20 OU, and IOP was stable at 21 mmHg OU.

## Discussion

We describe herein a case of TSPK treated using topical non-ophthalmic tacrolimus 0.1% ointment. A thorough patient history and detailed clinical evaluation are essential to establish the diagnosis and to exclude other causes of superficial punctate keratopathy, including viral keratitis, microsporidial keratoconjunctivitis, drug-induced keratopathy, and keratoconjunctivitis sicca [4]. Speculation on a post-viral cause has been made, given a possible association with recent viral infections. These include herpes simplex virus, varicella-zoster virus, or adenovirus, but no microbiological agent or virus has been identified as the definitive cause [3]. There is also a theory about a possible genetic link with the HLA-DR3 antigen, resulting in extended exacerbations and remissions. This antigen’s presence may potentially modify the immune response [4]. As this disease is uncommon, its exact origin remains a subject of debate. 

In TSPK, topical tacrolimus has previously been shown to improve symptoms such as tearing, photophobia, and foreign body sensation, as well as lead to the resolution of the corneal lesions; however, the use of this 0.1% concentration and ointment form has yet to be described. By suppressing T-cell activity, tacrolimus may reduce immune-mediated inflammation in the cornea, which is believed to contribute to lesion formation and recurrent symptoms in TSPK [4]. This provides a mechanistic rationale for its use in managing the condition.

In another case report, tacrolimus 0.02% drops, with a mean treatment duration of 10 weeks, were safe, well-tolerated, and effective in reducing ocular inflammation. All patients showed improvement in symptoms of tearing and photophobia, as well as a decrease in the number of lesions. Seven of these patients were unresponsive to topical corticosteroids and/or lubricants [6]. Tacrolimus ophthalmic ointment 0.03% was also effective for controlling TSPK with great patient tolerance and no noticeable local or systemic side effects. Patients who used the medication regularly experienced improved VA and symptom relief. Unfortunately, the treatment was not curative at a six-year follow-up [7].

In this case, we showed that topical non-ophthalmic tacrolimus 0.1% ointment can be an option for controlling TSPK with good patient tolerance and no noticeable local or systemic side effects. It showed a complete resolution of the lesions bilaterally with a nightly application for three weeks. Although lesions recurred after discontinuation of treatment, the patient remained asymptomatic throughout the 12-month follow-up and did not require restarting the tacrolimus. No complications were observed, and both IOP and VA remained stable. Tacrolimus appears to act as a steroid-sparing agent, potentially reducing the risk of steroid-induced IOP elevation [8]. 

Theoretically, a higher concentration could provide stronger immunosuppressive effects, which may have contributed to the persistent symptom relief observed in this patient. A longer course of treatment could potentially intensify this effect while also preventing lesion recurrence. The dissociation between signs and symptoms could also suggest a neurotrophic component, resulting in less perception of irritation despite the presence of lesions. Further studies are needed to evaluate the optimal concentration, duration, and balance between efficacy and safety. Higher concentrations could increase the risk of local irritation or reduce tolerability, which in this case may have been mitigated by the use of artificial tears.

Tacrolimus can be administered as eyedrops or ointment, with both formulations used in the literature. The choice of vehicle is often guided by patient comfort, as drops are generally easier to instill and cause less blurred vision, while ointments provide longer corneal contact and may reduce the frequency of application. Tacrolimus 0.1% in eyedrop form, administered two to four times daily with artificial tears, could potentially enhance patient comfort, ease of application, and allow more flexibility in titrating treatment. In this case report, the ointment was selected because it was more cost-effective and readily available compared to a compounded ophthalmic drop formulation. It was well tolerated by the patient when applied at night with artificial tears.

There are limitations associated with this report. It describes a single-patient observation, with a short duration of treatment (three-week course) and a 12-month follow-up, so the long-term outcomes of 0.1% tacrolimus in TSPK remain unknown. Long-term observation would be essential to determine whether complete remission can be achieved, as many patients have recurrences. Additionally, symptom improvement was based on the patient’s subjective report. Other objective measurements, including tear breakup time (TBUT), corneal sensitivity, standardized lesion grading, and symptom scoring using the Ocular Surface Disease Index (OSDI), were not assessed in this patient, as management was conducted in routine clinical care, which may limit the precision of signs and symptoms evaluation.

## Conclusions

Topical non-ophthalmic tacrolimus 0.1% ointment was effective in managing the signs and symptoms of TSPK in an adult patient. As the condition can exhibit a prolonged course with recurrences, tacrolimus is helpful as a steroid-sparing agent. It could be advantageous in avoiding IOP elevations secondary to steroid response while controlling symptoms and lesions in refractory patients. The higher 0.1% concentration may have played a role in the persistence of symptom relief even after cessation of treatment, although this observation is limited to a single patient. In contrast to the current treatment standard of topical corticosteroids, tacrolimus 0.1% combined with artificial tears may offer the advantage of causing fewer side effects when applied at night and could be considered a long-term option for symptomatic patients.
